# High-grade inflammation, metabolic abnormalities, and incident type 2 diabetes: a dynamic matched prospective cohort study

**DOI:** 10.3389/fendo.2026.1968030

**Published:** 2026-10-16

**Authors:** Yulong Lan, Dan Wu, Xiong Ding, Lingyu Xu, Hui Zhou, Mengyi Zheng, Zegui Huang, Peng Fu, Zekai Chen, Wei Wang, Shouling Wu, Youren Chen

**Affiliations:** 1 Department of Cardiology, The Second Affiliated Hospital of Shantou University Medical College, Shantou, China; 2 Nutrition and Health Innovation Research Institute, Edith Cowan University School of Medical and Health Sciences, Joondalup, WA, Australia; 3 Center for Endemic Disease Control, Chinese Center for Disease Control and Prevention, Harbin Medical University, Harbin, Heilongjiang, China; 4 Cardiovascular Medicine Division, University of Pennsylvania Perelman School of Medicine, Philadelphia, PA, United States; 5 Xiangya School of Nursing, Central South University, Changsha, Hunan, China; 6 Department of Cardiology, Beijing Chaoyang Hospital, Capital Medical University, Beijing, China; 7 Department of Cardiology, The First Affiliated Hospital of Dalian Medical University, Dalian, China; 8 Glycomedicine Research Institute, Shantou University Medical College, Shantou, Guangdong, China; 9 Department of Cardiology, Kailuan General Hospital, Tangshan, China

**Keywords:** high-grade inflammation, overweight and obesity, precision prevention, prospective cohort study, risk factor control, type 2 diabetes

## Abstract

**Aims:**

To investigate the association between high-grade inflammation and incident type 2 diabetes (T2D) and assess how this risk varied according to metabolic abnormalities and risk factor control.

**Methods:**

Within the Kailuan cohort, 9,347 individuals with high-grade inflammation (hsCRP ≥10 mg/L) between 2006/2007 and 2016/2017 were dynamically matched by age and sex at a prespecified 1:4 ratio to 31,584 non-inflamed participants. Multivariable Cox regression models adjusted for demographic, lifestyle, renal, lipid, blood pressure, and adiposity-related factors were used to estimate hazard ratios (HRs) for incident T2D overall, across metabolic and risk factor strata.

**Results:**

Participants with high-grade inflammation had greater adiposity and more adverse metabolic profiles than matched non-inflamed participants. During a median 10.1 years of follow-up, T2D developed in 17.7% of participants with high-grade inflammation and 13.1% of matched non-inflamed participants. High-grade inflammation was associated with a 24% higher adjusted risk of T2D (HR 1.24, 95% CI 1.16–1.33). Diabetes risks were further elevated when high-grade inflammation coexisted with metabolic abnormalities, ranging from 2.25 for overweight/obesity to 4.24 for impaired fasting glucose. Management of BMI, waist-to-height ratio, or blood pressure largely attenuated the excess risk, whereas favorable smoking or renal function control alone was insufficient.

**Conclusions:**

High-grade inflammation was associated with a higher risk of T2D, particularly in the presence of metabolic abnormalities. Maintaining a healthy weight and optimal blood pressure may help attenuate this risk, highlighting the potential importance of integrating high-grade inflammation and metabolic status in diabetes risk assessment and prevention.

## 1 Introduction

Type 2 diabetes affects more than 10% of the global population (1) and imposes a substantial burden on health systems worldwide (2, 3). Inflammation is a key driver of diabetogenesis, contributing to both insulin resistance and β-cell dysfunction (4, 5). While the role of chronic low-grade inflammation in diabetes development is well established, the impact of acute immune responses—reflected by markedly elevated inflammatory markers—remains poorly understood.

Emerging evidence suggests a potential link between acute immune challenges and the onset of type 2 diabetes. For instance, since the coronavirus disease 2019 (COVID-19) pandemic, type 2 diabetes has increasingly been recognized as a major metabolic sequela of severe acute respiratory syndrome coronavirus 2 (SARS-CoV-2) infection (6–9). Furthermore, observational studies indicate that viral infections and the associated immune activation are consistently related to impaired glucose regulation and higher diabetes risk (10–14). Notably, acute immune responses may accelerate diabetes onset in metabolically vulnerable individuals. For example, the incidence of diabetes was found to increase after SARS-CoV-2 infection, particularly among individuals with obesity, dyslipidemia, or insulin resistance (6). Experimental studies similarly demonstrate that acute infections accelerate diabetes progression in obese or insulin-resistant mice (15), while multi-omics analyses indicate impaired insulin signaling in insulin-resistant individuals exposed to acute viral infections (16). Collectively, these findings support a potential role of acute immune activation in diabetes pathogenesis, particularly through its interaction with metabolic dysfunction.

C-reactive protein (CRP), synthesized by the liver, is the most widely used biomarker of systemic inflammation (17). Numerous studies have shown that elevated CRP predicts diabetes risk, but most epidemiological evidence has focused on low-grade inflammation (hsCRP ≥2 (18, 19) or 3 mg/L (20–22)). In contrast, hsCRP concentrations ≥10 mg/L, commonly indicative of acute-phase responses (23), are typically excluded from analyses of cardiometabolic risk (17, 24, 25). A recent methodological commentary, however, has questioned this practice (26); nevertheless, the relevance of high-grade inflammation to diabetes risk, particularly in combination with metabolic disorders, remains uncertain.

To address this knowledge gap, we conducted a dynamic age- and sex-matched prospective cohort study within the Kailuan study in China. We aimed to investigate the association between high-grade inflammation (hsCRP ≥10 mg/L) and incident type 2 diabetes and to evaluate how this association varied according to metabolic abnormalities and risk factor control.

## 2 Methods

### 2.1 Study participants

The *Kailuan Study* (trial registration number: ChiCTR-TNC-11001489) has been previously described (27). It is an ongoing, real-world, prospective cohort study conducted in northern China and was approved by the Kailuan General Hospital Ethics Committee, China (2006–05). A total of 201,640 participants were recruited and underwent standardized biennial health examinations from 2006/2007 through 2020/2022.

The present substudy adopted a dynamic age- and sex-matched prospective cohort design. After excluding individuals with missing hsCRP data, pre-existing diabetes, or without follow-up information, 40,931 participants were included, comprising 9,347 participants with high-grade inflammation and 31,584 non-inflamed participants. At each eligible examination cycle, participants with high-grade inflammation were matched to non-inflamed participants from the corresponding examination cycle at a prespecified 1:4 ratio using the *GMATCH* macro (SAS 9.4), with exact matching on sex and age matched within a 2-year caliper. Matching was restricted to age and sex, with metabolic characteristics retained for subsequent multivariable and risk-stratified analyses, consistent with previous matched-cohort studies (28, 29). Age and sex were well balanced after matching, with standardized mean differences of 0.033 and 0.012, respectively. The participant selection and matching process is shown in **Supplementary Figure S1**.

### 2.2 Assessment of inflammation

Serum hsCRP was measured using a high-sensitivity nephelometric assay (Cias Latex CRP-H, Kanto Chemical Co. Inc., Tokyo, Japan). High-grade inflammation was defined as participants with CRP ≥10 mg/L, consistent with previous guidelines and literature indicating that concentrations at this level generally reflect marked or acute inflammatory activation (17, 23). The non-inflamed reference group was defined as hsCRP <1 mg/L, a threshold commonly used to indicate a low-inflammatory state (21, 30). Participants with hsCRP concentrations between 1 and <10 mg/L were excluded from the primary comparison because this range reflects low-grade inflammatory states and represents a different exposure contrast from high-grade inflammation versus a non-inflamed reference group. The median CRP level in our cohort (~100,000 participants per round; reported in **Supplementary Figure S1**) was consistently <1 mg/L, supporting the representativeness of the control group.

### 2.3 Assessment of outcomes

The primary outcome was the incidence of type 2 diabetes (International Classification of Diseases, 10th Revision [ICD-10]: E11), defined as fasting blood glucose (FBG) ≥7.0 mmol/L, a self-reported history of physician-diagnosed type 2 diabetes, or the use of oral antihyperglycemic agents with or without insulin therapy (31). During each examination, venous blood samples were collected from participants after a fast of at least 8 h, and FBG was measured using a Hitachi 7600 auto-analyzer (Hitachi, Tokyo, Japan) at the central laboratory of Kailuan General Hospital. The index date was defined as the qualifying examination at which participants entered the matched cohort during the health examination cycles conducted from 2006/2007 to 2016/2017. Follow-up began from this index examination. For participants who developed type 2 diabetes, the time-to-event was calculated from the index date to the date of diabetes onset. Participants who died without developing diabetes were censored at the date of death. Mortality status was continuously updated through linkage with medical insurance records and local governmental vital-status systems. Participants who remained alive and diabetes-free were censored at their last available follow-up examination.

### 2.4 Covariates

Included covariates were on the basis of current guideline (31), obtained at the enrollment health examination, including age, sex, lifestyle factors (smoking, alcohol use, physical activity), anthropometrics (height, weight, waist circumference, blood pressure), biochemical measures (triglycerides [TG], high-density lipoprotein cholesterol [HDL-C], creatinine), and family history of diabetes. Lifestyle factors were assessed according to the last year of the index visit (32), with smoking and drinking were classified as yes/no, and physical activity as active/inactive. Estimated glomerular filtration rate (eGFR) was calculated using the CKD-EPI equation. BMI (kg/m²) was categorized as underweight/normal, overweight, or obese according to Chinese (as the main analysis) and WHO criteria; fatty liver was assessed by ultrasonography; central obesity was defined as waist-to-height ratio (WHtR) ≥0.5; and impaired fasting glucose (IFG) as fasting blood glucose 6.1–6.9 mmol/L in non-diabetic individuals. Metabolic abnormalities included general obesity, central obesity, fatty liver, and IFG. Risk factor control was defined across four domains—systolic blood pressure (SBP) <130 mmHg, kidney function (eGFR ≥90 mL/min/1.73 m²), non-current smoking, and BMI <24 kg/m².

### 2.5 Statistical analysis

Baseline characteristics were summarized as median with interquartile range (IQR) for continuous variables and number (percentage) for categorical variables. The characteristics of raw data and missing values of covariates (generally <2.5%, except for physical activity [6.13%]) are shown in **Supplementary Table S1**. Missing data were handled by multiple imputation with chained equations (10 imputations) using the MI procedure in SAS. Imputation stability was assessed by comparing observed distributions with summary statistics across the 10 imputed datasets (**Supplementary Table S2**). Baseline differences between participants with high-grade inflammation and matched non-inflamed participants were assessed using the chi-square test for categorical variables and Mann–Whitney *U* test for continuous variables.

Incidence rates were calculated as the number of events divided by person-years at risk and expressed per 1,000 person-years. Cumulative hazard curves for type 2 diabetes were derived from Kaplan–Meier survival estimates, and between-group differences were assessed using the log-rank test. Multivariable Cox proportional hazards regression models, stratified on the matching identifier, were used to estimate hazard ratios (HRs) and 95% confidence intervals (CIs) for the association between high-grade inflammation and incident diabetes. The fully adjusted model included age, smoking status, alcohol consumption, physical activity, family history of diabetes, systolic blood pressure, log-transformed triglyceride-to-HDL-C ratio, eGFR, and BMI (Model 3). In alternative models, BMI was replaced by waist-to-height ratio (Model 4) or fatty liver degree (Model 5). The proportional hazards assumption was assessed using scaled Schoenfeld residuals, with diagnostic plots provided in the **Supplementary Figure S2**. Multiplicative interactions (INTm) were tested using the likelihood-ratio test with cross-product terms. Joint associations of high-grade inflammation and metabolic disorders with diabetes risk were also examined.

Sensitivity analyses included: (i) applying WHO BMI cutoffs, (ii) excluding diabetes cases occurring within the first 2 years of follow-up, and (iii) repeating analyses using unimputed data. To minimize potential confounding, additional analyses excluded participants with malignancy or cardiovascular disease (CVD) before diabetes onset, as well as those with pre-existing severely reduced kidney function (eGFR <30 mL/min/1.73 m²). Stratified analyses by age and sex were conducted to assess risk heterogeneity.

Finally, multivariable Cox proportional hazards regression models were used to evaluate the associations of overall and individual risk factor control with incident diabetes among participants with high-grade inflammation. Risk was examined according to the number of controlled factors as well as each individual factor.

All statistical tests were two-tailed, and significance was defined as *P* < 0.05. Analyses were performed using SAS version 9.4 (SAS Institute, Cary, NC, USA).

## 3 Results

**Table 1** summarizes baseline characteristics of the 40,931 participants, including 9,347 with high-grade inflammation and 31,584 matched non-inflamed participants. The median age was 52.0 years and 78.1% were men. Compared with the non-inflamed participants, participants with high-grade inflammation had slightly higher BMI, waist circumference, WHtR, systolic blood pressure, triglycerides, fasting glucose, and more frequent fatty liver, while reporting lower HDL-C and less physical activity; lifestyle factors were otherwise broadly comparable.

**Table 1:** Baseline characteristics of the study participants.

Characteristics	Total (n=40,931)	Non-inflamed participants (n=31,584)	High-grade inflammation participants (n=9347)	*P*-difference
Developing diabetes, No. (%)	5794.0 (14.2)	4140.0 (13.1)	1654.0 (17.7)	<0.01
Age, median (IQR), years	52.00 (43.00–59.00)	52.00 (43.00–59.00)	52.00 (43.00–60.00)	0.04
Male, No. (%)	31957 (78.1)	24624 (78.0)	7333.0 (78.5)	0.32
BMI, median (IQR), kg/m^2^	24.39 (22.31–26.70)	24.22 (22.10–26.37)	25.27 (23.05–27.69)	<0.01
BMI-categories				<0.01
BMI<24	18179 (44.4)	14891 (47.1)	3288.0 (35.2)	
24≤BMI<28	16700 (40.8)	12752 (40.4)	3948.0 (42.2)	
BMI≥28	6052.0 (14.8)	3941.0 (12.5)	2111.0 (22.6)	
Waist circumference, median (IQR), cm	86.00 (80.00–92.00)	85.00 (80.00–91.00)	88.00 (82.00–95.00)	<0.01
WhtR, median (IQR)	0.51 (0.48–0.55)	0.51 (0.47–0.54)	0.53 (0.49–0.56)	<0.01
Fatty liver degree, No. (%)	13043 (31.9)	9135 (28.9)	3908 (41.8)	<0.01
SBP, median (IQR), mm Hg	128.33 (117.33–140.00)	127.33 (116.67–140.00)	130.00 (120.00–140.67)	<0.01
HDL-C, median (IQR), mmol/L	1.44 (1.22–1.71)	1.45 (1.24–1.72)	1.39 (1.17–1.66)	<0.01
TG, median (IQR), mmol/L	1.20 (0.84–1.75)	1.19 (0.84–1.73)	1.22 (0.85–1.83)	<0.01
FBG, median (IQR), mmol/L	5.13 (4.73–5.60)	5.12 (4.73–5.57)	5.18 (4.72–5.66)	<0.01
eGFR, median (IQR), mL/min/1.73 m²	89.91 (73.94–103.41)	90.20 (73.85–103.66)	89.10 (74.11–102.65)	0.05
Education, No. (%)				0.26
Less than high school	30083 (73.5)	23171 (73.4)	6912 (73.9)	
High school and above	10848 (26.5)	8413 (26.6)	2435 (26.1)	
Current drinker, No. (%)	14472 (35.4)	11247 (35.6)	3225 (34.5)	0.05
Current smoker, No. (%)	26682 (65.2)	20618 (65.3)	6064 (64.9)	0.47
Physical activities, No. (%)				<0.01
Low	8539 (20.9)	6545 (20.7)	1994 (21.3)	
Moderate	26171 (63.9)	20094 (63.6)	6077 (65.0)	
High	6221.0 (15.2)	4945.0 (15.7)	1276 (13.7)	
Family history of diabetes, No. (%)	3025.0 (7.39)	2281.0 (7.22)	744 (7.96)	0.02

BMI, body mass index; eGFR, estimated glomerular filtration rate; HDL-C, high-density lipoprotein cholesterol; hsCRP, high-sensitivity C-reactive protein; LDL-C, low-density lipoprotein cholesterol; SBP, systolic blood pressure; TC, total cholesterol; TG, triglyceride; WHtR, waist-to-height ratio.

During a median follow-up of 10.1 (IQR 7.1–12.2) years, diabetes developed in 17.7% of participants with high-grade inflammation and 13.1% of matched non-inflamed participants. High-grade inflammation was associated with a higher incidence rate (18.71 vs 13.81 per 1,000 person-years) and a 24% greater diabetes risk after full adjustment (HR 1.24, 95% CI: 1.16–1.33), consistent with cumulative hazard curves (**Figure 1**). The findings were unchanged after additional adjustment for WHtR or fatty liver. BMI-stratified analyses showed heterogeneous associations (*P*-interaction = 0.042). The strongest relative risk was observed in participants with BMI 24–28 kg/m² (HR 1.35, 95% CI: 1.20–1.53), with a weaker but significant association in those with BMI <24 kg/m² (HR 1.31, 95% CI: 1.11–1.57). By contrast, although obese participants (BMI ≥28 kg/m²) had the highest absolute incidence rates, the association with high-grade inflammation was not significant (HR 0.92, 95% CI: 0.73–1.15) (**Table 2**). The association remained significant across binary strata of BMI, central obesity, and fatty liver but not within IFG (**Supplementary Tables S3–S6**).

**Figure 1:**
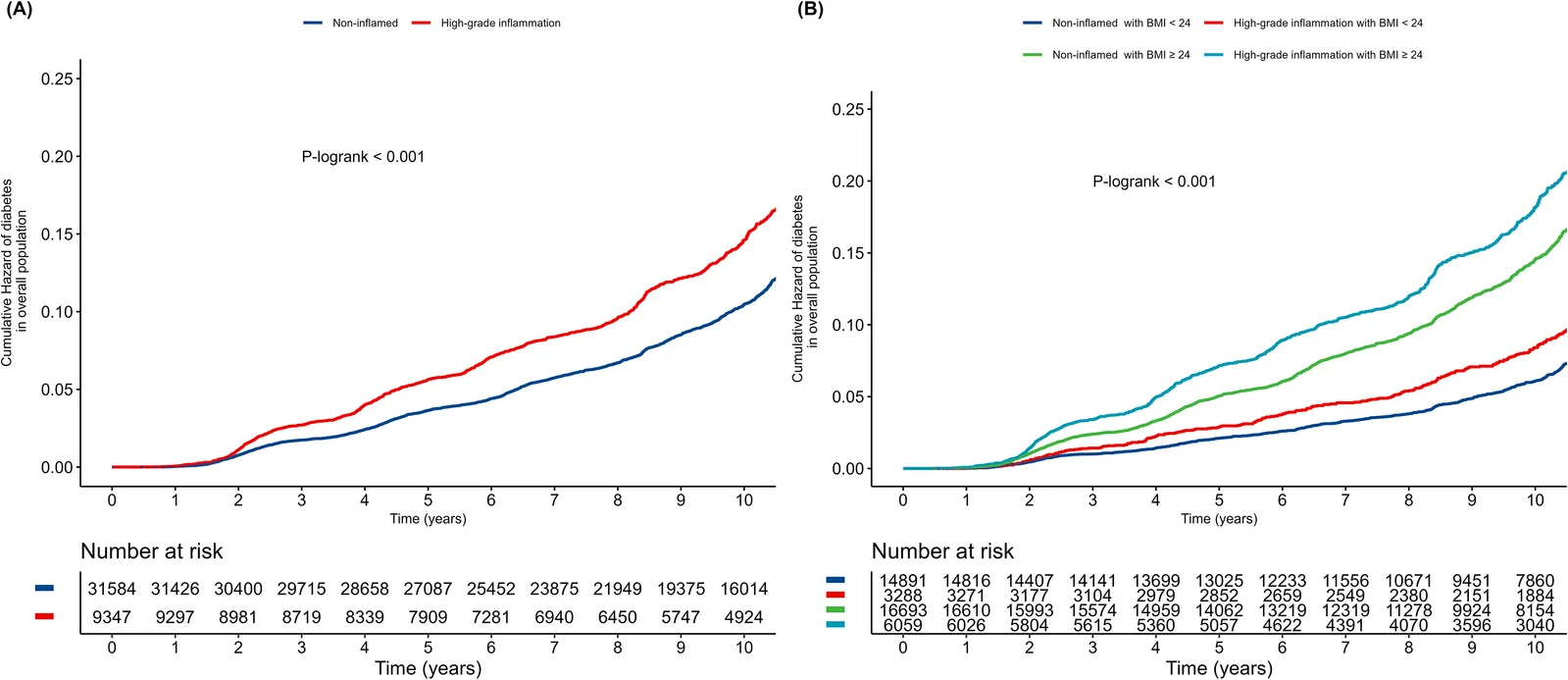
Cumulative hazards of type 2 diabetes according to high-grade inflammation in entire individuals and across BMI status. **(A)** Kaplan–Meier curves of cumulative hazards of type 2 diabetes among participants with high-grade inflammation (hsCRP ≥10 mg/L) compared with age- and sex-matched non-inflamed participants. Participants with high-grade inflammation consistently showed higher cumulative hazards over the 10-year follow-up (log-rank *P* < 0.001). **(B)** Kaplan–Meier curves stratified by BMI category (<24 vs ≥24 kg/m²) and inflammation status. The excess diabetes risk associated with high-grade inflammation was particularly pronounced among participants with overweight/obesity (BMI ≥24 kg/m²), who exhibited the steepest cumulative hazards (log-rank *P* < 0.001).

**Table 2:** The risk of incident type 2 diabetes upon high-grade inflammation in entire participants and cross different BMI statuses.

	Associations between high-grade inflammation and incident diabetes, HR (95% CI)		
Model/Subgroup	Non-inflamed	High-grade inflammation	P-trend
Event/Total	4140/31584	1654/9347	
Incidence rate	13.81 (13.39–14.23)	18.71 (17.83–19.63)	
Crude model	Ref.	1.43 (1.34–1.53)	<0.001
Model 1	Ref.	1.43 (1.34–1.52)	<0.001
Model 2	Ref.	1.35 (1.26–1.44)	<0.001
Model 3	Ref.	1.24 (1.16–1.33)	<0.001
Model 4	Ref.	1.24 (1.16–1.33)	<0.001
Model 5	Ref.	1.23 (1.15–1.32)	<0.001
In BMI<24 kg/m^2^			
Event/Total	1221/14891	360/3288	
Incidence rate	8.48 (8.02–8.97)	11.16 (10.06–12.37)	
Crude model	Ref.	1.35 (1.14–1.60)	<0.001
Model 1	Ref.	1.33 (1.12–1.58)	<0.001
Model 2	Ref.	1.31 (1.11–1.57)	0.002
In 24≤BMI<28 kg/m^2^			
Event/Total	1977/12753	755/3948	
Incidence rate	16.53 (15.81–17.27)	20.45 (19.05–21.97)	
Unadjusted model	Ref.	1.37 (1.21–1.54)	<0.001
Model 1	Ref.	1.37 (1.22–1.55)	<0.001
Model 2	Ref.	1.35 (1.20–1.53)	<0.001
In BMI≥28 kg/m^2^			
Event/Total	1977/3941	539/2111	
Incidence rate	26.01 (24.39–27.72)	28.03 (25.76–30.49)	
Crude model	Ref.	0.94 (0.75–1.18)	0.608
Model 1	Ref.	0.94 (0.75–1.17)	0.568
Model 2	Ref.	0.92 (0.73–1.15)	0.456

*P*-INTm: BMI (<24, 24-28, ≥28 kg/m^2^) * CRP matched case-controls =0.042.

Model 1: adjusted for age (continuous), smoking habits (yes or no), alcohol consumption (yes or no), physical activities (active or inactive), family history of diabetes (yes or no).

Model 2: further adjusted for SBP (continuous) and *log*(TG/HDL-C) (continuous), eGFR (continuous) on the basis of Model 1.

Model 3: additionally adjusted for BMI on the basis of Model 2.

Model 4: additionally adjusted for WHtR on the basis of Model 2.

Model 5: additionally adjusted for fatty liver degree on the basis of Model 2.

The incidence rate is per 1,000 person-years.

BMI, body mass index; INTm, multiplicative interaction; others as in **Table 1**.

When considering BMI as exposure, overweight/obesity (BMI ≥24 kg/m²) was associated with a higher risk of incident T2D compared with BMI <24, both overall and within inflammation strata. The adjusted HRs were 1.68 (95% CI, 1.53–1.84) among non-inflamed participants and 1.79 (95% CI, 1.18–2.71) among participants with high-grade inflammation, with no statistically significant multiplicative interaction (**Supplementary Table S7**).

Co-exposure analyses demonstrated that participants with both metabolic disorders and high-grade inflammation consistently had the highest incidence and diabetes risk (**Table 3**). Compared with non-inflamed, normal counterparts, risks were greatest for combined exposure to BMI ≥24 (HR 2.25, 95% CI: 2.04–2.47), central obesity (HR 1.93, 95% CI: 1.76–2.13), fatty liver (HR 2.38, 95% CI: 2.15–2.62), or IFG (HR 4.24, 95% CI: 3.64–4.94). Cumulative hazard curves further illustrated that co-occurrence of high-grade inflammation with metabolic disorders conferred the steepest cumulative hazards of diabetes, followed by those with either condition alone, consistent with the regression results (**Figure 1**, **Supplementary Figure S3**). Stratifying BMI into three categories further showed a progressive gradient, with the greatest risk in obese participants with high-grade inflammation (**Supplementary Table S8**). Results were consistent when using WHO BMI cut-offs (**Supplementary Tables S9, S10**). Sensitivity analyses, excluding early diabetes cases, participants with pre-existing CVD, cancer or severely reduced renal function, together with complete-case analyses using unimputed data, yielded similar findings (**Supplementary Table S11**). Subgroup analyses showed no heterogeneity by age (<55 vs ≥55 years; *P*-interaction = 0.614) but a stronger effect among women (*P*-interaction = 0.014; **Supplementary Tables S12, S13**).

**Table 3:** The risk of incident type 2 diabetes upon coexposure to metabolic abnormalities and high-grade inflammation.

Co-exposure to metabolic disorders and high-grade inflammation, HRs (95% CIs)					P-trend
Model	BMI<24 & non-inflamed	BMI<24 & high-grade inflammation	BMI≥24 & non-inflamed controls	BMI≥24 & high-grade inflammation	
Event/Total	1221/14891	360/3288	2919/16693	1294/6059	
Incident rate	8.48 (8.02–8.97)	11.16 (10.06–12.37)	18.73 (18.07–19.42)	23.05 (21.83–24.34)	
Crude model	Ref.	1.36 (1.18–1.56)	2.17 (2.00–2.35)	2.85 (2.59–3.12)	<0.001
Model 1	Ref.	1.34 (1.17–1.54)	2.14 (1.98–2.32)	2.82 (2.57–3.09)	<0.001
Model 2	Ref.	1.32 (1.15–1.53)	1.76 (1.62–1.91)	2.25 (2.04–2.47)	<0.001
	Non-central obesity & non-inflamed	Non-central obesity & high-grade inflammation	Central obesity & non-inflamed	Central obesity & high-grade inflammation	
Event/Total	1194/13694	333/2841	2946/17890	1321/6506	
Incident rate	9.03 (8.53–9.55)	12.31 (11.06–13.71)	17.58 (16.96–18.23)	21.53 (20.40–22.72)	<0.001
Crude model	Ref.	1.44 (1.25–1.66)	1.82 (1.68–1.98)	2.39 (2.18–2.62)	<0.001
Model 1	Ref.	1.42 (1.23–1.64)	1.81 (1.67–1.96)	2.38 (2.17–2.61)	<0.001
Model 2	Ref.	1.36 (1.18–1.58)	1.51 (1.38–1.64)	1.93 (1.76–2.13)	<0.001
	Non-fatty liver & non-inflamed	Non-fatty liver & high-grade inflammation	Fatty liver & non-inflamed	Fatty liver & high-grade inflammation	
Event/Total	2210/22449	694/5439	1930/9135	960/3908	
Incident rate	10.05 (9.63–10.47)	12.90 (11.98–13.90)	24.18 (23.12–25.28)	27.72 (26.02–29.53)	
Crude model	Ref.	1.33 (1.21–1.47)	2.41 (2.23–2.61)	2.93 (2.67–3.22)	<0.001
Model 1	Ref.	1.33 (1.20–1.46)	2.40 (2.22–2.60)	2.92 (2.66–3.21)	<0.001
Model 2	Ref.	1.30 (1.18–1.44)	1.97 (1.81–2.14)	2.38 (2.15–2.62)	<0.001
	Non-IFG & non-inflamed	Non-IFG & high-grade inflammation	IFG & non-inflamed	IFG & high-grade inflammation	
Event/Total	3105/28903	1222/8308	1035/2681	432/1039	
Incident rate	11.21 (10.82–11.61)	15.26 (14.43–16.14)	45.32 (42.64–48.16)	51.72 (47.07–56.84)	
Crude model	Ref.	1.46 (1.35–1.57)	4.60 (4.15–5.10)	4.87 (4.20–5.66)	
Model 1	Ref.	1.45 (1.35–1.57)	4.61 (4.16–5.12)	4.85 (4.18–5.64)	
Model 2	Ref.	1.39 (1.29–1.50)	4.24 (3.81–4.71)	4.24 (3.64–4.94)	

Model 1: adjusted for age (continuous), smoking habits (yes or no), alcohol consumption (yes or no), physical activities (active or inactive), family history of diabetes (yes or no).

Model 2: further adjusted for SBP (continuous) and *log* (TG/HDL-C) (continuous), eGFR (continuous) on the basis of Model 1.

Risk factor control analyses revealed that participants with high-grade inflammation who met all four favorable risk factor criteria (SBP, BMI, smoking, eGFR) had the lowest diabetes incidence and a lower risk of incident diabetes than the overall non-inflamed reference group (HR 0.60, 95% CI: 0.46–0.78). Risk increased stepwise with fewer controlled factors, reaching nearly two-fold higher risk with no control (HR 1.93, 95% CI: 1.65–2.25). Similar gradients were observed when restricting to participants with high-grade inflammation, with risk rising from HR 1.62 (95% CI: 1.21–2.18) for three controlled factors to HR 3.95 (95% CI: 2.88–5.44) with none (**Figure 2**, **Supplementary Tables S14, S15**). Specific risk factor analyses further showed that control of BMI, WHtR, or SBP was protective and largely eliminated the excess diabetes risk (HRs approaching null). In particular, BMI control was associated with a significant protective effect (HR 0.85, 95% CI: 0.76–0.95). By contrast, smoking control conferred only a modest benefit, while eGFR and eGFR control did not meaningfully reduce diabetes risk (**Figure 3**, **Supplementary Table S16**).

**Figure 2:**
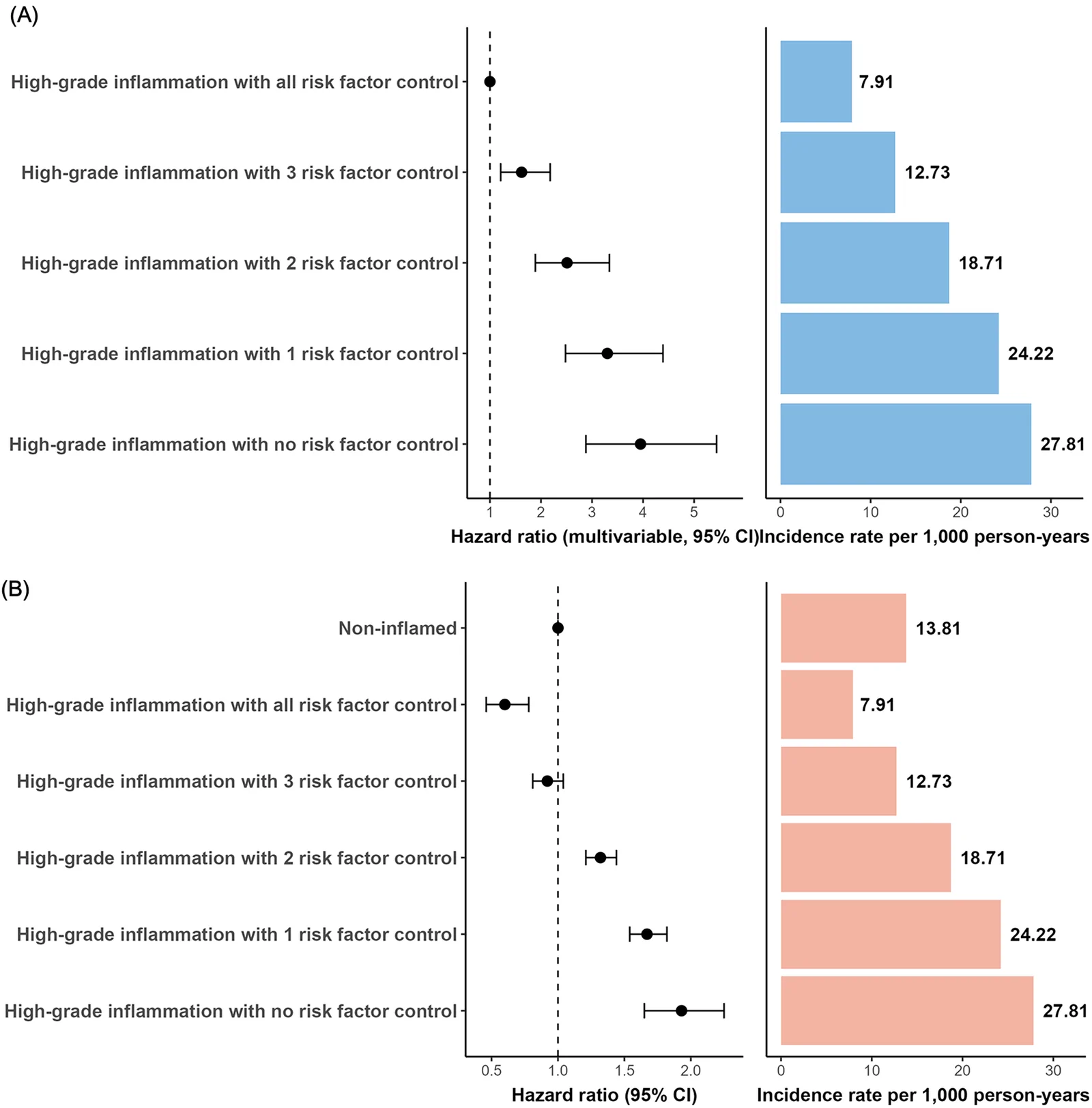
Risk of incident type 2 diabetes according to the level of risk factor management among participants with high-grade inflammation. **(A)** Risk factor control and incident type 2 diabetes among participants with high-grade inflammation. Incidence rates and hazard ratios of type 2 diabetes are shown according to the number of risk factors meeting the prespecified control criteria (BMI, SBP, smoking, eGFR). Diabetes risk declined progressively as the number of controlled risk factors increased. **(B)** Risk factor control and incident type 2 diabetes among participants with high-grade inflammation, compared with matched non-inflamed inflammation. Participants with high-grade inflammation who met all four risk factor control criteria had the lowest diabetes incidence and a lower risk than the matched non-inflamed reference group, whereas fewer controlled risk factors were associated with progressively higher diabetes risk.

**Figure 3:**
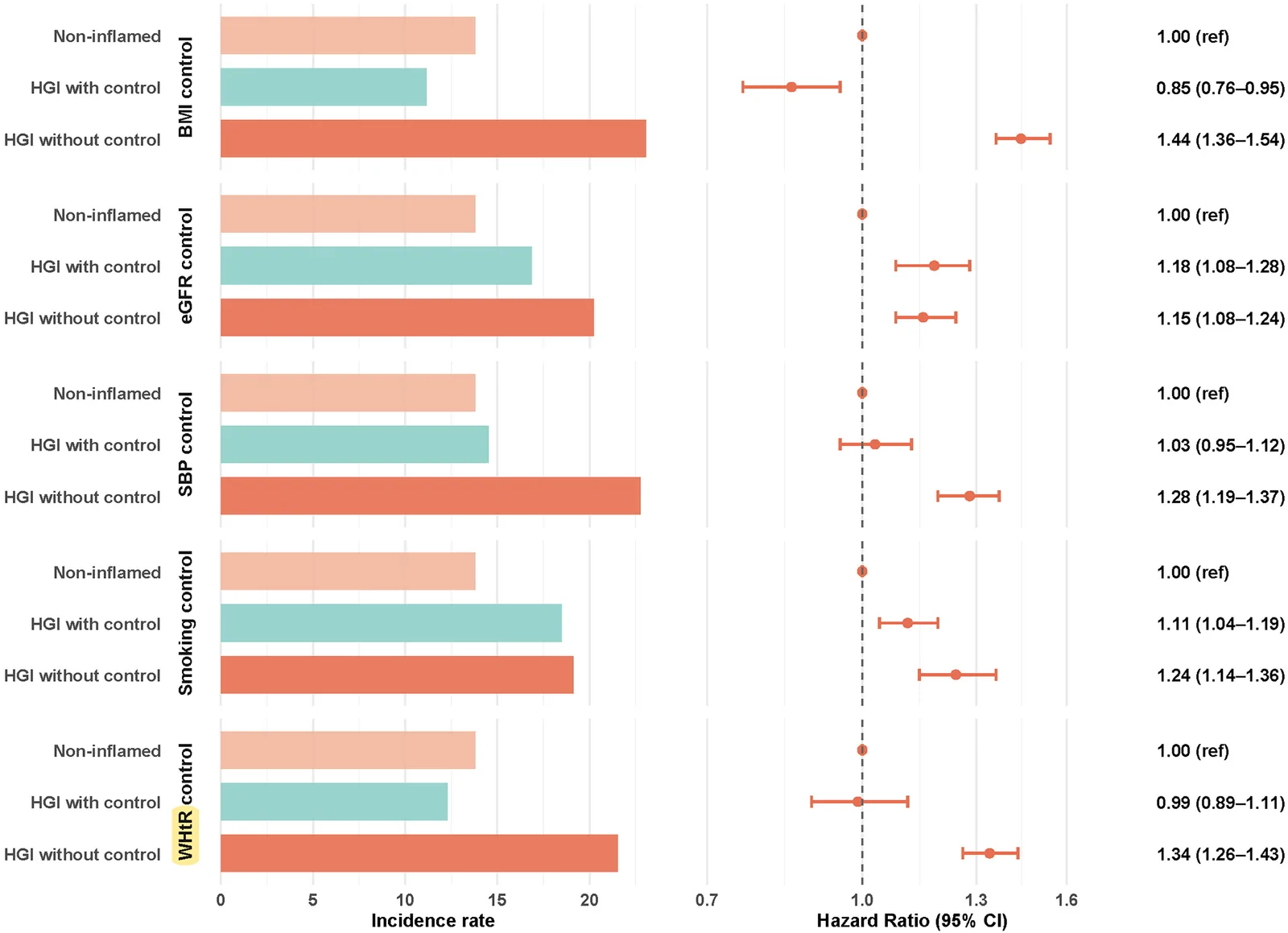
Risk of incident type 2 diabetes according to specific risk factor control among participants with high-grade inflammation compared with matched non-inflamed participants. Incidence rates are expressed as diabetes events per 1,000 person-years. multivariable-adjusted hazard ratios (HRs) with 95% confidence intervals (CIs) were estimated using Cox proportional hazards models. Multivariable models were adjusted for age (continuous), alcohol consumption (yes or no), physical activity (active or inactive), family history of diabetes (yes or no), and log-transformed TG/HDL-C ratio, as well as BMI (continuous, except for BMI control analysis), SBP (continuous, except for SBP control analysis), eGFR (continuous, except for eGFR control analysis), and smoking status (yes or no, except for smoking control analysis). Matched non-inflamed participants served as the reference group.

## 4 Discussion

Drawing on a dynamic, age- and sex-matched, prospective cohort study within the real-life prospective Kailuan cohort, we showed that high-grade inflammation (hsCRP ≥10 mg/L) was significantly associated with incident type 2 diabetes after multivariable adjustment, including adjustment for BMI, corresponding to a 24% higher risk. This risk was further elevated when metabolic abnormalities coexisted, including overweight/obesity, central adiposity, fatty liver, and impaired fasting glucose, and declined progressively with better risk factor control, particularly for adiposity and blood pressure.

We observed effect heterogeneity by BMI: relative risks were most pronounced in participants with normal to moderately elevated BMI, whereas the association was less evident among those with obesity, possibly reflecting a ceiling effect of high baseline metabolic risk. Nonetheless, co-exposure analyses confirmed that the absolute diabetes incidence and diabetes risk were greatest when high-grade inflammation and metabolic abnormalities coexisted. The progressively higher risks observed with adiposity-related abnormalities, fatty liver, and particularly IFG likely reflect increasing metabolic vulnerability. IFG represents a more advanced stage of dysglycemia, whereas adiposity and fatty liver are closely linked to insulin resistance and metabolic dysfunction. However, because these conditions were assessed concurrently at baseline, our data cannot establish a temporal causal sequence among them. These findings are compatible with the hypothesis that acute immune challenges may accelerate diabetogenesis in metabolically vulnerable individuals through mechanisms involving insulin resistance, β-cell dysfunction, and amplification of adipose tissue inflammation (33).

Notably, our findings provide epidemiological evidence that high-grade inflammation is associated with subsequent risk of type 2 diabetes. While most previous studies have focused on low-grade inflammation, our work suggests that high-grade inflammatory states may also be relevant to diabetes risk, particularly in individuals with underlying metabolic vulnerability. CRP, predominantly synthesized by the liver, remains relatively stable under basal conditions, whereas marked elevations commonly occur during acute immune challenges (17). Although hsCRP is a powerful risk biomarker for incident diabetes, it should be regarded as a marker rather than a causal agent (30). Consistent with this interpretation, experimental studies have shown that neither inhibition of CRP production with antisense oligonucleotides (34) nor infusion of pharmaceutical-grade CRP (35) altered systemic inflammation or metabolic outcomes. Thus, markedly elevated hsCRP may reflect an acute inflammatory state that is associated with increased diabetes risk, particularly when metabolic abnormalities coexist.

Although hsCRP ≥10 mg/L can arise from diverse conditions such as infections, trauma, cancer, or acute stress (17, 30), infectious and other acute immune challenges represent biologically plausible contributors to marked inflammatory responses. This is consistent with emerging evidence linking infectious diseases (e.g., COVID-19, cytomegalovirus, human immunodeficiency virus, hepatitis C) to higher diabetes incidence (6–14, 36). Mechanistically, acute inflammatory cytokine signaling may impair insulin action through stress-responsive pathways that disrupt insulin receptor substrate–PI3K–Akt signaling, thereby promoting peripheral insulin resistance. In parallel, pathogen-related inflammation may induce oxidative and endoplasmic-reticulum stress in pancreatic β-cells, impair insulin secretion, and, under more severe conditions, contribute to β-cell dysfunction or death (15, 37–40). These effects may be particularly consequential in individuals with obesity or pre-existing insulin resistance, in whom metabolic compensatory capacity is already limited. Taken together, high-grade inflammatory responses may act as a metabolic stressor or trigger that increases diabetes risk in metabolically susceptible individuals, rather than acting through hsCRP itself as a causal mediator. Further diabetes-focused mechanistic studies are warranted to elucidate the pathways through which high-grade inflammatory responses interact with metabolic vulnerability to promote progression toward diabetes.

Importantly, our study highlights the relevance of risk factor control among participants with high-grade inflammation. Diabetes risk increased progressively as fewer risk factor criteria were met. Favorable BMI and blood pressure profiles were associated with the greatest attenuation of excess diabetes risk, whereas favorable smoking or renal function status alone was insufficient to achieve comparable risk reduction. These findings highlight the potential importance of weight and blood pressure management in individuals experiencing high-grade inflammatory states.

### 4.1 Clinical implications

Our findings underscore several key clinical messages. First, high-grade inflammation should not be simply excluded as an “acute response” but recognized as a meaningful risk state for diabetes onset. Second, the substantially higher risk observed when high-grade inflammation coexisted with metabolic abnormalities highlights the importance of considering inflammatory and metabolic status together in risk assessment. Third, favorable adiposity and blood pressure profiles were associated with marked attenuation of the excess diabetes risk among participants with high-grade inflammation, supporting the potential value of weight and blood pressure management in diabetes prevention. Given that acute inflammatory or immune events are often unpredictable, long-term maintenance of favorable metabolic health may help reduce vulnerability to subsequent diabetes development.

This study has several notable strengths. First, to our knowledge, it is the first dynamic age- and sex-matched prospective cohort study to investigate the association between high-grade inflammation and incident type 2 diabetes. Second, we systematically assessed the joint associations of high-grade inflammation and metabolic abnormalities with incident T2D, showing that risk was greatest when both were present. Third, the analysis of risk factor control across adiposity, blood pressure, renal function, and smoking provided clinically relevant information on risk attenuation among participants with high-grade inflammation. Finally, the large-scale, prospective Kailuan cohort with standardized data collection and rigorous quality assurance enhanced the validity and reliability of our findings.

Several limitations should be acknowledged. First, participants were primarily Han Chinese drawn largely from an industrial workforce in northern China, which may limit the generalizability of our findings to other regions of China and to populations with different ethnic, socioeconomic, or occupational backgrounds. Second, hsCRP was assessed at a single index examination, and therefore transient acute-phase elevations could not be distinguished from more sustained high-grade inflammatory states. Third, because health examinations were conducted biennially, continuous monitoring of acute immune events and short-term fluctuations in hsCRP was not possible, and some transient responses may have been missed. Additionally, markedly elevated hsCRP can arise from diverse conditions, including infection, trauma, stress, or malignancy, and the specific cause of elevation could not be determined in individual participants. Moreover, information on several other chronic inflammatory or immune-related conditions, including chronic obstructive pulmonary disease, chronic liver disease, primary immunodeficiencies, human immunodeficiency virus/acquired immunodeficiency syndrome, and corticosteroid use, was not systematically available. Nevertheless, sensitivity analyses excluding participants with cancer, CVD, or severely reduced kidney function yielded similar findings. Despite these limitations, our study provides epidemiological evidence that high-grade inflammation is associated with subsequent diabetes risk and highlights the potential value of maintaining favorable metabolic health.

### 4.2 Conclusion

In this large, dynamic matched prospective cohort, high-grade inflammation (hsCRP ≥10 mg/L) was associated with a 24% higher risk of incident type 2 diabetes. The risk was further elevated in the presence of metabolic abnormalities but decreased progressively with better risk factor control. Notably, favorable BMI and blood pressure profiles were associated with substantial attenuation of the excess risk related to high-grade inflammation, highlighting the potential importance of weight and blood pressure management in diabetes prevention.

## Data Availability

The raw data supporting the conclusions of this article will be made available by the authors, without undue reservation.
