# Supplementary material for: High-grade inflammation, metabolic abnormalities, and incident type 2 diabetes: a dynamic matched prospective cohort study
Source: Front Endocrinol (Lausanne). 2026 Oct 16;17:1968030. doi: 10.3389/fendo.2026.1968030 (PMC13719346; doi:10.3389/fendo.2026.1968030)
Supplement: Supplementary file 1 [file SupplementaryFile1.docx]

**Supplementary Materials**

[Table S1. Baseline characteristics of the study participants (raw data)	2](#_Toc240212374)

[Table S2. Comparison of observed and imputed distributions across 10 multiply imputed datasets	4](#_Toc240212375)

[Table S3. The risk of incident type 2 diabetes upon high-grade inflammation in entire participants	5](#_Toc240212376)

[Table S4. The risk of incident type 2 diabetes upon high-grade inflammation across central obesity	6](#_Toc240212377)

[Table S5. The risk of incident type 2 diabetes upon high-grade inflammation across fatty liver status	7](#_Toc240212378)

[Table S6. The risk of incident type 2 diabetes upon high-grade inflammation across IFG status	8](#_Toc240212379)

[Table S7. The risk of incident type 2 diabetes upon overweight across inflammation status	9](#_Toc240212380)

[Table S8. The risk of incident type 2 diabetes upon co-exposure to (normal weight, overweight and obesity, Chinese criteria) and high-grade inflamamtion	10](#_Toc240212381)

[Table S9. The risk of incident type 2 diabetes upon co-exposure to overweight/obesity (WHO criteria) and high-grade inflammation	11](#_Toc240212382)

[Table S10. The risk of incident type 2 diabetes upon co-exposure to BMI strata (normal weight, overweight and obesity, WHO criteria) and high-grade inflammation	13](#_Toc240212383)

[Table S11. Sensitivity analysis risk of incident type 2 diabetes upon co-exposure to overweight/obesity and high-grade inflammation	15](#_Toc240212384)

[Table S12. Age-stratified analysis risk of incident type 2 diabetes upon co-exposure to overweight/obesity and high-grade inflammation	16](#_Toc240212385)

[Table S13. Sex-stratified analysis risk of incident type 2 diabetes upon co-exposure to overweight/obesity and high-grade inflammation	17](#_Toc240212386)

[Table S14. The risk of incident type 2 diabetes upon prevailing risk factor in entire participants	18](#_Toc240212387)

[Table S15. The risk of incident type 2 diabetes upon prevailing risk factor in participants in high-grade inflammation	19](#_Toc240212388)

[Table S16. The effect of specific risk factor control in high-grade inflammation participants on incident diabetes compared to no-inflamed individuals	20](#_Toc240212389)

[Figure S1. Flowchart of participant recruitment and dynamic age- and sex-matched cohort formation	21](#_Toc240212390)

[Figure S2. Assessment of the proportional hazards assumption using scaled Schoenfeld residuals	23](#_Toc240212391)

[Figure S3. Cumulative hazards of type 2 diabetes across joint high-grade inflammation and metabolic abnormalities	25](#_Toc240212392)

# Table S1. Baseline characteristics of the study participants (raw data)

|  | **Total** | **Non-inflamed participants** | **Among high-grade inflammation participants** |
| --- | --- | --- | --- |
| Age, median (IQR), years | 52.00 (43.00–59.00) | 52.00 (43.00–59.00) | 52.00 (43.00–60.00) |
| Male, No. (%) | 31957 (78.1) | 24624 (78.0) | 7333.0 (78.5) |
| BMI, median (IQR), kg/m^2^,  (MissingN=96 [0.23%]) | 24.39 (22.32–26.70) | 24.22 (22.10–26.37) | 25.27 (23.05–27.69) |
| Fatty liver, No. (%),  (MissingN=25 [0.06%]) | 12938 39.1) | 9131 (28.9) | 3907.0 (41.8) |
| SBP, median (IQR), mm Hg  (MissingN=453 [1.11%]) | 128.33 (117.70–140.00) | 127.33 (116.67–140.00) | 130.00 (120.00–140.67) |
| Waist circumferece, median (IQR), cm  (MissingN=252 [0.62%]) | 86.00 (80.00–92.00) | 85.00 (80.00–91.00) | 88.00 (82.00–95.00) |
| HDL-C, median (IQR), mmol/L  (MissingN=56 [0.14%]) | 1.44 (1.22–1.71) | 1.45 (1.24–1.72) | 1.39 (1.17–1.66) |
| TG, median (IQR), mmol/L  (MissingN=73 [0.18%]) | 1.20 (0.84–1.75) | 1.19 (0.84–1.73) | 1.22 (0.85–1.83) |
| eGFR, median (IQR), mL/min/1.73 m²  (MissingN=105 [0.26%]) | 89.91 (73.94–103.41) | 90.21 (73.86–103.66) | 89.10 (74.10–102.62) |
| Family history of diabetes, No. (%) | 3025.0 (7.39) | 2281.0 (7.22) | 744.00 (7.96) |
| Education, No. (%)  (MissingN=466 [1.14%]) |  |  |  |
| Less than high school | 29755 (73.5) | 22924 (73.4) | 6831.0 (73.9) |
| High school and above | 10710 (26.5) | 8301.0 (26.6) | 2409.0 (26.1) |
| Drinking habits, No. (%)  (MissingN=982 [2.40%]) |  |  |  |
| Yes | 25891 (64.8) | 19909 (64.6) | 5982.0 (65.5) |
| No | 14058 (35.2) | 10911 (35.4) | 3147.0 (34.5) |
| Smoking habits, No. (%)  (MissingN=114 [0.28%]) |  |  |  |
| Never and ever smoker | 26605 (65.2) | 20573 (65.3) | 6032.0 (64.8) |
| Current smoker | 14212 (34.8) | 10942 (34.7) | 3270.0 (35.2) |
| Physical activity, No. (%)  (MissingN=2511 [6.13%]) |  |  |  |
| Low | 7981.0 (20.8) | 6103.0 (20.6) | 1878.0 (21.3) |
| Moderate | 24543 (63.9) | 18797 (63.5) | 5746.0 (65.1) |
| High | 5896.0 (15.3) | 4693.0 (15.9) | 1203.0 (13.6) |

Abbreviations: BMI: body mass index; eGFR: estimated glomerular filtration rate; HDL-C: high-density lipoprotein cholesterol; hsCRP: high-sensitivity C-reactive protein; LDL-C: low-density lipoprotein cholesterol; missingN: number of missing variables; SBP: systolic blood pressure; TC: total cholesterol; TG: triglyceride.

# Table S2. Comparison of observed and imputed distributions across 10 multiply imputed datasets

| **Variable** | **Missing, n (%)** | **Observed distribution** | **Average summary statistic across**  **10 imputed datasets** | **Range across 10 imputed datasets** |
| --- | --- | --- | --- | --- |
| BMI, kg/m² | 96 (0.23) | 24.39 (22.32–26.70) | 24.38 (22.31–26.71) | 24.38–24.39 |
| SBP, mmHg | 453 (1.11) | 128.33 (117.70–140.00) | 128.34 (117.71–140.02) | 128.31–128.35 |
| Waist circumference, cm | 252 (0.62) | 86.00 (80.00–92.00) | 86.02 (80.05–92.00) | 86.00–86.03 |
| HDL-C, mmol/L | 56 (0.14) | 1.44 (1.22–1.71) | 1.44 (1.22–1.71) | 1.44–1.44 |
| TG, mmol/L | 73 (0.18) | 1.20 (0.84–1.75) | 1.21 (0.84–1.74) | 1.20–1.21 |
| eGFR, mL/min/1.73 m² | 105 (0.26) | 89.91 (73.94–103.41) | 89.92 (73.95–103.42) | 89.91–89.93 |
| Fatty liver, % | 25 (0.06) | 39.1 | 39.1 | 39.1–39.2 |
| Education, ≥high school, % | 466 (1.14) | 26.5 | 26.5 | 26.4–26.6 |
| Current drinking, % | 982 (2.40) | 64.8 | 64.9 | 64.7–65.0 |
| Current smoking, % | 114 (0.28) | 34.8 | 34.8 | 34.8–34.9 |
| Moderate/high physical  activity, % | 2511 (6.13) | 79.2 | 79.1 | 78.9–79.3 |

Continuous variables are presented as median (interquartile range [IQR]), and categorical variables as percentages. The observed distribution was calculated using available non-missing values in the original dataset before multiple imputation. Summary statistics were calculated separately in each of the 10 multiply imputed datasets. The average of these dataset-specific summary statistics is presented in the fourth column. For continuous variables, the range represents the minimum to maximum of the dataset-specific medians across the 10 imputed datasets; for categorical variables, it represents the minimum to maximum of the dataset-specific percentages.

# Table S3. The risk of incident type 2 diabetes upon high-grade inflammation in entire participants

|  | **Non-inflamed** | **High-grade inflammation** | ***P*-trend** |
| --- | --- | --- | --- |
| Among participants with BMI<24 kg/m^2^ | | | |
| Event/Total | 1221/14891 | 360/3288 |  |
| Incidence rate | 8.48 (8.02‒8.97) | 11.16 (10.06‒12.37) |  |
| Crude model | Ref. | 1.35 (1.14‒1.60) | <0.001 |
| Model 1 | Ref. | 1.33 (1.12‒1.58) | <0.001 |
| Model 2 | Ref. | 1.31 (1.11‒1.57) | 0.002 |
| Among participants with BMI>24 kg/m^2^ | | | |
| Event/Total | 2919/16693 | 1294/6059 |  |
| Incidence rate | 18.73 (18.07‒19.42) | 23.05 (21.83‒24.34) |  |
| Crude model | Ref. | 1.29 (1.18‒1.40) | <0.001 |
| Model 1 | Ref. | 1.29 (1.19‒1.41) | <0.001 |
| Model 2 | Ref. | 1.26 (1.15‒1.37) | <0.001 |
| *P*-INTm: 0.684 | | | |

Model 1: adjusted for sex (male or female), age (continuous), smoking habits (yes or no), alcohol consumption (yes or no), physical activities (active or inactive), family history of diabetes (yes or no).

Model 2: further adjusted for SBP (continuous) and *log*(TG/HDL-C) (continuous), eGFR (continuous) on the basis of Model 1.

# Table S4. The risk of incident type 2 diabetes upon high-grade inflammation across central obesity

|  | **Non-inflamed** | **High-grade inflammation** | ***P*-trend** |
| --- | --- | --- | --- |
| Among participants with non-central obesity | | | |
| Event/Total | 1194/13694 | 333/2841 |  |
| Incidence rate | 9.03 (8.53‒9.55) | 12.31 (11.06‒13.71) |  |
| Crude model | Ref. | 1.49 (1.24‒1.79) | <0.001 |
| Model 1 | Ref. | 1.47 (1.22‒1.77) | <0.001 |
| Model 2 | Ref. | 1.42 (1.17‒1.72) | <0.001 |
| Among participants with central obesity | | | |
| Event/Total | 2946/17890 | 1321/6506 |  |
| Incidence rate | 17.58 (16.95‒18.23) | 21.53 (20.40‒22.72) |  |
| Crude model | Ref. | 1.31 (1.21‒1.42) | <0.001 |
| Model 1 | Ref. | 1.31 (1.21‒1.42) | <0.001 |
| Model 2 | Ref. | 1.29 (1.19‒1.40) | <0.001 |
| *P*-INTm: 0.502 | | | |

Model 1: adjusted for sex (male or female), age (continuous), smoking habits (yes or no), alcohol consumption (yes or no), physical activities (active or inactive), family history of diabetes (yes or no).

Model 2: further adjusted for SBP (continuous) and *log*(TG/HDL-C) (continuous), eGFR (continuous) on the basis of Mode 1.

# Table S5. The risk of incident type 2 diabetes upon high-grade inflammation across fatty liver status

|  | **Non-inflamed** | **High-grade inflammation** | ***P*-trend** |
| --- | --- | --- | --- |
| Among participants with non-fatty liver | | | |
| Event/Total | 2210/22449 | 694/5439 |  |
| Incidence rate | 10.05 (9.63‒10.47) | 12.90 (11.98‒13.90) |  |
| Crude model | Ref. | 1.33 (1.20‒1.48) | <0.001 |
| Model 1 | Ref. | 1.32 (1.19‒1.47) | <0.001 |
| Model 2 | Ref. | 1.28 (1.15‒1.43) | <0.001 |
| Among participants with fatty liver | | | |
| Event/Total | 1930/9135 | 960/3908 |  |
| Incidence rate | 24.18 (23.12‒25.28) | 27.72 (26.03‒29.53) |  |
| Crude model | Ref. | 1.13 (1.00‒1.27) | 0.055 |
| Model 1 | Ref. | 1.13 (1.00‒1.28) | 0.050 |
| Model 2 | Ref. | 1.12 (0.99‒1.27) | 0.063 |
| *P*-INTm: 0. 325 | | | |

Model 1: adjusted for sex (male or female), age (continuous), smoking habits (yes or no), alcohol consumption (yes or no), physical activities (active or inactive), family history of diabetes (yes or no).

Model 2: further adjusted for SBP (continuous) and *log*(TG/HDL-C) (continuous), eGFR (continuous) on the basis of Model 1.

# Table S6. The risk of incident type 2 diabetes upon high-grade inflammation across IFG status

|  | **Non-inflamed participants** | **Among high-grade inflammation participants** | ***P*-trend** |
| --- | --- | --- | --- |
| Among individuals without IFG | | | |
| Event/Total | 3105/28903 | 1222/8308 |  |
| Incidence rate | 11.21 (10.82‒11.61) | 15.26 (14.43‒16.14) |  |
| Crude model | Ref. | 1.46 (1.35‒1.57) | <0.001 |
| Model 1 | Ref. | 1.46 (1.35‒1.57) | <0.001 |
| Model 2 | Ref. | 1.38 (1.28‒1.50) | <0.001 |
| Among individuals with IFG | | | |
| Event/Total | 1035/2681 | 432/1039 |  |
| Incidence rate | 45.32 (42.64‒48.16) | 51.72 (47.07‒58.84) |  |
| Crude model | Ref. | 0.93 (0.69‒1.25) | 0.627 |
| Model 1 | Ref. | 0.92 (0.68‒1.24) | 0.582 |
| Model 2 | Ref. | 0.90 (0.66‒1.21) | 0.484 |
| *P*-INTm: 0.0010 | | | |

Model 1: adjusted for sex (male or female), age (continuous), smoking habits (yes or no), alcohol consumption (yes or no), physical activities (active or inactive), family history of diabetes (yes or no).

Model 2: further adjusted for SBP (continuous) and *log*(TG/HDL-C) (continuous), eGFR (continuous) on the basis of Model 1.

Abbreviation: IFG, impaired fasting glucose.

# Table S7. The risk of incident type 2 diabetes upon overweight across inflammation status

|  | **BMI<24 kg/m^2^** | **BMI≥24 kg/m^2^** | ***P*-trend** |
| --- | --- | --- | --- |
| In whole population | | | |
| Event/Total | 3105/18179 | 1222/22752 |  |
| Incidence rate | 8.97 (8.54‒9.42) | 19.87 (19.28‒20.48) |  |
| Crude model | Ref. | 2.21 (2.06‒2.38) | <0.001 |
| Model 1 | Ref. | 2.20 (2.05‒2.36) | <0.001 |
| Model 2 | Ref. | 1.78 (1.66‒1.92) | <0.001 |
| Among non-inflamed participants | | | |
| Event/Total | 1221/14891 | 2919/16693 |  |
| Incidence rate | 8.48 (8.02‒8.97) | 18.73 (18.07‒19.42) |  |
| Crude model | Ref. | 2.10 (1.92‒2.28) | <0.001 |
| Model 1 | Ref. | 2.07 (1.90‒2.26) | <0.001 |
| Model 2 | Ref. | 1.68 (1.53‒1.84) | <0.001 |
| Among Among high-grade inflammation participants | | | |
| Event/Total | 360/3288 | 1294/6059 |  |
| Incidence rate | 11.16 (10.06‒12.37) | 23.05 (21.83‒24.34) |  |
| Crude model | Ref. | 2.05 (1.41‒2.98) | <0.001 |
| Model 1 | Ref. | 2.21 (1.50‒3.28) | <0.001 |
| Model 2 | Ref. | 1.79 (1.18‒2.71) | 0.006 |
| *P*-INTm: 0.684 | | | |

Model 1: adjusted for sex (male or female), age (continuous), smoking habits (yes or no), alcohol consumption (yes or no), physical activities (active or inactive), family history of diabetes (yes or no).

Model 2: further adjusted for SBP (continuous) and *log*(TG/HDL-C) (continuous), eGFR (continuous) on the basis of Model 1

# Table S8. The risk of incident type 2 diabetes upon co-exposure to (normal weight, overweight and obesity, Chinese criteria) and high-grade inflamamtion

|  | **Normal weight & non-inflamed** | **Overweight & non-inflamed** | **Obesity & non-inflamed** | **Normal weight & high-grade inflammation** | **Overweight & high-grade inflammation** | **Obesity & high-grade inflammation** |
| --- | --- | --- | --- | --- | --- | --- |
| Event/Total | 1221/14891 | 1977/12753 | 1977/3941 | 360/3288 | 755/3948 | 539/2111 |
| Incident rate | 8.48 (8.02‒8.97) | 16.53 (15.81‒17.27) | 26.01 (24.39‒27.72) | 11.16 (10.06‒12.37) | 20.45 (19.05‒21.97) | 28.03 (25.76‒30.49) |
| Crude model | Ref. | 1.86 (1.71‒2.03) | 3.28 (2.94‒3.67) | 1.35 (1.18‒3.55) | 2.52 (2.26‒2.81) | 3.52 (3.09‒4.01) |
| Model 1 | Ref. | 1.84 (1.69‒2.01) | 3.23 (2.90‒3.61) | 1.34 (1.16‒3.54) | 2.50 (2.24‒2.79) | 3.48 (3.05‒3.96) |
| Model 2 | Ref. | 1.58 (1.44‒1.72) | 2.45 (2.18‒2.75) | 1.32 (1.15‒1.52) | 2.10 (1.88‒2.35) | 2.62 (2.29‒3.00) |

Model 1: adjusted for age (continuous), smoking habits (yes or no), alcohol consumption (yes or no), physical activities (active or inactive), family history of diabetes (yes or no), antihypertensives, and lipid-lowering drugs.

Model 2: further adjusted for SBP (continuous), eGFR (continuous) and *log*(TG/HDL-C) (continuous) on the basis of Model 1.

# Table S9. The risk of incident type 2 diabetes upon co-exposure to overweight/obesity (WHO criteria) and high-grade inflammation

|  | **Co-exposure to overweight/obesity (WHO criteria) and high-grade inflammation, HRs (95% CIs)** | | | | ***P*-trend** |
| --- | --- | --- | --- | --- | --- |
|  | **BMI<25 & non-inflamed** | **BMI<25 & high-grade inflammation** | **BMI≥25 & non-inflamed** | **BMI≥25 & high-grade inflammation** |  |
| Event/Total | 1766/18908 | 546/4385 | 2374/12676 | 1108/4962 |  |
| Incident rate | 9.73 (9.29‒10.20) | 12.83 (11.80‒13.96) | 20.06 (19.27‒20.88) | 24.16 (22.78‒25.62) |  |
| Crude model | Ref. | 1.39 (1.24‒1.55) | 2.08 (1.93‒2.24) | 2.64 (2.41‒2.89) | <0.001 |
| Model 1 | Ref. | 1.38 (1.24‒1.55) | 2.06 (1.91‒2.22) | 2.62 (2.39‒2.86) | <0.001 |
| Model 2 | Ref. | 1.35 (1.21‒1.52) | 1.69 (1.56‒1.83) | 2.10 (1.91‒2.31) | <0.001 |

Model 1: adjusted for age (continuous), smoking habits (yes or no), alcohol consumption (yes or no), physical activities (active or inactive), family history of diabetes (yes or no), antihypertensives, and lipid-lowering drugs.

Model 2: further adjusted for SBP (continuous), eGFR (continuous) and *log*(TG/HDL-C) (continuous) on the basis of Model 1.

# Table S10. The risk of incident type 2 diabetes upon co-exposure to BMI strata (normal weight, overweight and obesity, WHO criteria) and high-grade inflammation

|  | **Normal weight & non-inflamed** | **Overweight & non-inflamed** | **Obesity & non-inflamed** | **Normal weight & high-grade inflammation** | **Overweight & high-grade inflammation** | **Obesity & high-grade inflammation** |
| --- | --- | --- | --- | --- | --- | --- |
| Event/Total | 1766/18908 | 1978/11183 | 396/1493 | 546/4385 | 827/3978 | 281/984 |
| Incident rate | 9.73 (9.29–10.20) | 18.88 (18.06–19.73) | 29.19 (26.46–32.22) | 12.83 (11.80–13.96) | 22.29 (20.82–23.86) | 32.07 (28.53–36.04) |
| Crude model | Ref. | 1.95 (1.80‒2.11) | 3.15 (2.73‒3.65) | 1.38 (1.24‒1.55) | 2.40 (2.17‒2.66) | 3.74 (3.15‒4.44) |
| Model 1 | Ref. | 1.93 (1.79‒2.09) | 3.11 (2.69‒3.60) | 1.38 (1.23‒1.54) | 2.37 (2.15‒2.63) | 3.72 (3.13‒4.42) |
| Model 2 | Ref. | 1.61 (1.49‒1.75) | 2.36 (2.03‒2.75) | 1.35 (1.21‒1.52) | 1.97 (1.78‒2.19) | 2.73 (2.28‒3.26) |

Model 1: adjusted for age (continuous), smoking habits (yes or no), alcohol consumption (yes or no), physical activities (active or inactive), family history of diabetes (yes or no), antihypertensives, and lipid-lowering drugs.

Model 2: further adjusted for SBP (continuous), eGFR (continuous) and *log*(TG/HDL-C) (continuous) on the basis of Model 1

# Table S11. Sensitivity analysis risk of incident type 2 diabetes upon co-exposure to overweight/obesity and high-grade inflammation

| **Co-exposure to overweight/obesity and high-grade inflammation, HRs (95% CIs)** | | | | |
| --- | --- | --- | --- | --- |
|  | **BMI<24 & non-inflamed** | **BMI<24 & high-grade inflammation** | **BMI≥24 & non-inflamed** | **BMI≥24 & high-grade inflammation** |
| **Excluded diabetes occurred in the first two years** | | | | |
| Incident rate | 8.06 (7.61–8.54) | 10.65 (9.58–11.84) | 17.76 (17.11–18.44) | 21.71 (20.52–22.97) |
| Multivariable model | Ref. | 1.33 (1.15–1.54) | 1.77 (1.62–1.93) | 2.24 (2.03–2.48) |
| **Excluded participants with cancer before diabetes onset** | | | | |
| Incident rate | 8.56 (8.09–9.06) | 11.25 (10.13–12.49) | 18.84 (18.16–19.54) | 23.08 (21.83–24.39) |
| Multivariable model | Ref. | 1.32 (1.14–1.52) | 1.76 (1.61–1.91) | 2.24 (2.03–2.47) |
| **Excluded participants with cardiovascular disease before diabetes onset** | | | | |
| Incident rate | 8.42 (7.94–8.92) | 11.06 (9.92–12.33) | 18.89 (18.19–19.61) | 23.23 (21.93–24.61) |
| Multivariable model | Ref. | 1.34 (1.16–1.56) | 1.78 (1.63–1.95) | 2.34 (2.11–2.60) |
| **Excluded participants with severely reduced renal dysfunction before diabetes onset** | | | | |
| Incident rate | 8.48 (8.02–8.97) | 11.17 (10.07–12.38) | 18.72 (18.06–19.42) | 23.06 (21.83–24.35) |
| Multivariable model | Ref. | 1.31 (1.14–1.51) | 1.76 (1.62–1.92) | 2.25 (2.04–2.48) |
| **Excluded participants with incomplete covariates** | | | | |
| Incident rate | 8.48 (8.02‒8.97) | 11.16 (10.06‒12.37) | 18.73 (18.07‒19.42) | 23.05 (21.83‒24.34) |
| Multivariable model | Ref. | 1.35 (1.17–1.56) | 1.78 (1.63–1.95) | 2.31 (2.09–2.56) |

Severely reduced renal dysfunction was indicated by eGFR <30 mL/min/1.73 m².

Multivariable models were adjusted for age (continuous), smoking habits (yes or no), alcohol consumption (yes or no), physical activities (active or inactive), family history of diabetes (yes or no), SBP (continuous), eGFR (continuous) and *log*(TG/HDL-C) (continuous).

# Table S12. Age-stratified analysis risk of incident type 2 diabetes upon co-exposure to overweight/obesity and high-grade inflammation

| **Co-exposure to overweight/obesity and high-grade inflammation, HRs (95% CIs)** | | | | | ***P*-INTm** |
| --- | --- | --- | --- | --- | --- |
|  | **BMI<24 & non-inflamed participants** | **BMI<24 & high-grade inflammation** | **BMI≥24 & non-inflamed** | **BMI≥24 & high-grade inflammation** |  |
| **<55 years** | | | | | **0.614** |
| Incident rate | 7.86(7.30–8.46) | 10.39(9.05–11.92) | 17.62(16.78–18.50) | 22.86(21.29–24.56) |  |
| Multivariable model | Ref. | 1.23(1.02–1.48) | 1.65(1.47–1.84) | 2.29(2.01–2.60) |  |
| **≥55 years** | | | | |  |
| Incident rate | 9.53(8.74–10.39) | 12.32(10.54–14.40) | 20.29(19.22–21.41) | 23.31(21.43–25.36) |  |
| Multivariable model | Ref. | 1.46(1.18–1.81) | 1.91(1.68–2.17) | 2.20(1.89–2.55) |  |

Multivariable models were adjusted for smoking habits (yes or no), alcohol consumption (yes or no), physical activities (active or inactive), family history of diabetes (yes or no), SBP (continuous), eGFR (continuous) and *log*(TG/HDL-C) (continuous).

# Table S13. Sex-stratified analysis risk of incident type 2 diabetes upon co-exposure to overweight/obesity and high-grade inflammation

| **Co-exposure to overweight/obesity and high-grade inflammation, HRs (95% CIs)** | | | | | ***P*-INTm** |
| --- | --- | --- | --- | --- | --- |
|  | **BMI<24 & non-inflamed** | **BMI<24 & high-grade inflammation** | **BMI≥24 & non-inflamed** | **BMI≥24 & high-grade inflammation** |  |
| **In males** | | | | | **0.014** |
| Incident rate | 9.32(8.75–9.92) | 11.43(10.18–12.84) | 18.93(18.19–19.70) | 23.02(21.64–24.48) |  |
| Multivariable model | Ref. | 1.23 (1.05–1.44) | 1.68 (1.53–1.85) | 2.14 (1.92–2.39) |  |
| **In females** | | | | |  |
| Incident rate | 6.18 (5.44–7.02) | 10.22 (8.15–12.81) | 17.85 (16.38–19.45) | 23.17 (20.61–26.03) |  |
| Multivariable model | Ref. | 1.76 (1.29–2.39) | 2.09 (1.73–2.52) | 2.64 (2.14–3.26) |  |

Multivariable models were adjusted for age (continuous), smoking habits (yes or no), alcohol consumption (yes or no), physical activities (active or inactive), family history of diabetes (yes or no), SBP (continuous), eGFR (continuous) and *log*(TG/HDL-C) (continuous).

# Table S14. The risk of incident type 2 diabetes upon prevailing risk factor in entire participants

|  | **Non-inflamed participants** | **High-grade inflammation participants with all risk factor control** | **High-grade inflammation participants with 3 risk factor control** | **High-grade inflammation participants with 2 risk factor control** | **High-grade inflammation participants with 1 risk factor control** | **Hgh-grade inflammation participants with no risk factor control** |
| --- | --- | --- | --- | --- | --- | --- |
| Incidence rate | 13.81 (13.39‒14.23) | 7.91 (6.05–10.36) | 12.73 (11.27–14.39) | 18.71 (17.24–20.31) | 24.22 (22.37–26.23) | 27.81 (23.91–32.35) |
| Crude model | Ref. | 0.56 (0.43–0.74) | 0.90 (0.79–1.02) | 1.36 (1.24–1.48) | 1.77 (1.62–1.92) | 2.01 (1.72–2.35) |
| Multivariable model | Ref. | 0.60 (0.46–0.78) | 0.92 (0.81–1.04) | 1.32 (1.21–1.44) | 1.67 (1.54–1.82) | 1.93 (1.65–2.25) |

Risk factor control including SBP (<130 mmHg), BMI (<24 kg/m^2^), non-smoking, and eGFR≥90 mL/min/1.73 m².

Multivariable model was adjusted for sex (male or female), age (continuous), alcohol consumption (yes or no), physical activities (active or inactive), family history of diabetes (yes or no) and log (TG/HDL-C) (continuous).

Incidence rates are expressed as events per 1,000 person-years.

Non-inflamed participants served as the reference group.

# Table S15. The risk of incident type 2 diabetes upon prevailing risk factor in participants in high-grade inflammation

|  | **High-grade inflammation with all risk factor control** | **High-grade inflammation with 3 risk factor control** | **High-grade inflammation with 2 risk factor control** | **High-grade inflammation with 1 risk factor control** | **High-grade inflammation with no risk factor control** |
| --- | --- | --- | --- | --- | --- |
| Incidence rate | 7.91(6.05–10.36) | 12.73(11.27–14.39) | 18.71(17.24–20.31) | 24.22(22.37–26.23) | 27.81(23.91–32.35) |
| Crude model | Ref. | 1.59 (1.18–2.13) | 2.40(1.81–3.18) | 3.11(2.35–4.12) | 3.56(2.61–4.84) |
| Multivariable model | Ref. | 1.62(1.21–2.18) | 2.51(1.89–3.34) | 3.30(2.48–4.39) | 3.95(2.88–5.44) |

Risk factor control including SBP (<130 mmHg), BMI (<24 kg/m^2^), non-smoking, and eGFR ≥90 mL/min/1.73 m².

Multivariable model was adjusted for sex (male or female), age (continuous), alcohol consumption (yes or no), physical activities (active or inactive), family history of diabetes (yes or no), log (TG/HDL-C) (continuous).

Incidence rates are expressed as events per 1,000 person-years.

High-grade inflammation participants with all risk factors control served as the reference group.

# Table S16. The effect of specific risk factor control in high-grade inflammation participants on incident diabetes compared to no-inflamed individuals

|  | **Among non-inflamed participants** | **High-grade inflammation with control** | **High-grade inflammation without control** | ***P*-trend** |
| --- | --- | --- | --- | --- |
| **BMI control** |  |  |  |  |
| Incidence rate | 13.81 (13.39–14.23) | 11.16 (10.06–12.37) | 23.05 (21.83–24.34) |  |
| Crude model | Ref. | 0.78 (0.70–0.87) | 1.69 (1.59–1.80) | <0.001 |
| Multivariable-model | Ref. | 0.85 (0.76–0.95) | 1.44 (1.36–1.54) | <0.001 |
| **WHtR control** |  |  |  |  |
| Incidence rate | 13.81 (13.39–14.23) | 12.31 (11.06–13.71) | 21.53 (20.40–22.72) |  |
| Crude model | Ref. | 0.88 (0.79–0.99) | 1.55 (1.46–1.65) | <0.001 |
| Multivariable-model | Ref. | 0.99 (0.89–1.11) | 1.34 (1.26–1.43) | <0.001 |
| **SBP control** |  |  |  |  |
| Incidence rate | 13.81 (13.39–14.23) | 14.51 (13.42–15.69) | 22.76 (21.41–24.20) |  |
| Crude model | Ref. | 1.02 (0.94–1.11 | 1.67 (1.56–1.79) | <0.001 |
| Multivariable-model | Ref. | 1.03 (0.95–1.12) | 1.28 (1.19–1.37) | <0.001 |
| **Smoking control** |  |  |  |  |
| Incidence rate | 13.81 (13.39–14.23) | 18.48 (17.41–19.63) | 19.13 (17.65–20.74) |  |
| Crude model | Ref. | 1.32 (1.23–1.41) | 1.41 (1.29–1.53) | <0.001 |
| Multivariable-model | Ref. | 1.11 (1.04–1.19) | 1.24 (1.14–1.36) | <0.001 |
| **eGFR control** |  |  |  |  |
| Incidence rate | 13.81 (13.39–14.23) | 16.86 (15.63–18.18) | 20.24 (19.01–21.55) |  |
| Crude model | Ref. | 1.26 (1.16–1.37) | 1.42 (1.32–1.52) | <0.001 |
| Multivariable-model | Ref. | 1.18 (1.08–1.28) | 1.15 (1.08–1.24) | <0.001 |

Incidence rates are expressed as events per 1,000 person-years.

Multivariable models were adjusted for sex (male or female), age (continuous), alcohol consumption (yes or no), physical activity (active or inactive), family history of diabetes (yes or no), and log-transformed TG/HDL-C ratio, as well as BMI (continuous, except for BMI control analysis), SBP (continuous, except for SBP control analysis), eGFR (continuous, except for eGFR control analysis), and smoking status (yes or no, except for smoking control analysis).

Non-inflamed participants served as the reference group.

# Figure S1. Flowchart of participant recruitment and dynamic age- and sex-matched cohort formation

**Caption**: A total of 201,604 participants attended the biennial health examinations between 2006/2007 and 2020/2022. After excluding 42,630 individuals without hsCRP measurements and 73,510 individuals without follow-up information on diabetes after the occurrence of high-grade inflammation, 85,464 participants remained eligible. Of these, 11,615 individuals with high-grade inflammation were identified. After further excluding 44,533 participants with missing data on sex/age or pre-existing diabetes, 40,931 participants were included in the matched case–control study (9,347 high-grade inflammation participants and 31,584 contemporary non-inflamed participants). Participants and participants were matched in a 1:4 ratio by age (caliper ≤2 years) and sex using the *GMATCH* macro in SAS.

# Figure S2. Assessment of the proportional hazards assumption using scaled Schoenfeld residuals

**Caption**: Scaled Schoenfeld residual plots for high-grade inflammation and covariates included in the primary Cox proportional hazards regression model. The solid line represents the smoothed residual pattern over follow-up time, and the dashed lines indicate the corresponding 95% confidence bands. No substantial deviation from the proportional hazards assumption was observed based on visual inspection of the scaled Schoenfeld residuals.

# Figure S3. Cumulative hazards of type 2 diabetes across joint high-grade inflammation and metabolic abnormalities

**Caption**: In the BMI-stratified analysis (**Panel A**), participants with both overweight/obesity (BMI ≥25 kg/m²) and high-grade inflammation had the steepest cumulative hazard curve, followed by those with either condition alone, while the lowest hazard was observed in non-inflamed participants with BMI <25 kg/m².

Similar dose–response patterns were observed for central obesity (**Panel B**), fatty liver (**Panel C**) and impaired fasting glucose ([IFG]; **Panel D**), where individuals with combined high-grade inflammation and metabolic abnormalities showed the highest hazards, with intermediate risks for those with single conditions. All differences across subgroups were statistically significant (log-rank *P* <0.001).
